# Clinical features of primary and compound forms of wide macular posterior staphyloma in high myopia

**DOI:** 10.1186/s12886-024-03519-1

**Published:** 2024-06-11

**Authors:** Chang Ki Yoon, Eun Kyoung Lee, Kunho Bae, Un Chul Park

**Affiliations:** Department of Ophthalmology, Seoul National University College of Medicine, 103 Daehak-ro, Jongno-gu, Seoul, 110-799 Korea

**Keywords:** Choroidal thickness, Myopic maculopathy, Pathologic myopia, Posterior staphyloma, Scleral thickness, Widefield optical coherence tomography

## Abstract

**Background:**

To compare the ocular features of highly myopic eyes with posterior staphyloma of wide macular type according to its morphological complexity.

**Methods:**

In this cross-sectional study, wide macular posterior staphyloma (WMPS) was classified into the primary (Curtin type I) and the compound (Curtin types VI to X) forms based on the configuration within the staphyloma. The grades of myopic maculopathy and the thicknesses of choroid and sclera were compared between the primary and compound forms of WMPS.

**Results:**

A total of 154 eyes (103 patients) with primary WMPS and 65 eyes (49 patients) with compound WMPS were included. Eyes with compound WMPS had worse visual acuity (*P* = 0.001) and greater axial length (*P* < 0.001) than those with primary WMPS. Compared to primary WMPS, compound WMPS had a higher grade of myopic macular degeneration (*P* < 0.001) and a higher frequency of lamellar or full-thickness macular hole associated with myopic traction (21.5% vs. 10.4%; *P* = 0.028) and active or scarred myopic choroidal neovascularization (33.8% vs. 20.1%; *P* = 0.030). On swept-source optical coherence tomography, eyes with compound WMPS had significantly thinner choroid and sclera.

**Conclusions:**

The compound form of WMPS had more severe myopic macular changes and worse visual prognosis compared to the primary form of WMPS, and these were associated with more structural deformation in the posterior eyeball. Compound WMPS should be considered as an advanced form of staphyloma.

## Background

Pathologic myopia is one of the leading etiologies of irreversible vision loss worldwide, especially in East Asia where the prevalence of myopia is high [1–3]. Two main ocular structural alterations in pathologic myopia are axial elongation and posterior staphyloma [4], which indicates localized outpouching of the eyeball wall with a radius of curvature smaller than the surrounding region [5]. Highly myopic eyes with posterior staphyloma are reported to have significantly lower visual acuity and more frequent chorioretinal atrophic change, myopic choroidal neovascularization (CNV), and myopic traction maculopathy than those without staphyloma [5, 6]. 

In 1977, Curtin classified posterior staphylomas into 10 different types based on ophthalmoscopic appearance [7]. Types I to V were termed primary staphylomas, but the remaining types VI to X were considered compound staphylomas because they appeared to be composites of more than one type of staphyloma or elaboration from primary staphyloma. Recently, applying 3D-MRI and ultra-widefield fundus images, Ohno-Matsui simplified Curtin’s classification into six types and renamed them according to their location and distribution: wide macular type, narrow macular type, peripapillary type, nasal type, inferior type, and others [5]. With the difficulty in reflecting various kinds of scleral irregularities within the staphyloma, only the outermost border of the staphyloma was considered in the classification. Thus, five types (VI to X) of compound staphylomas, for which Curtin described as mostly the variants of the primary type I staphyloma [7], were included under the same category as type I and renamed as the wide macular type. However, the precise prevalence of the compound form of wide macular posterior staphyloma (WMPS) and its clinical features compared to the primary form have not been reported yet, although the compound form seems to account for a considerable proportion of WMPS.

Scleral irregularities within the compound form of WMPS (Curtin types VI to X), which usually result from the combination of two or more types of primary staphyloma, may represent non-uniform or selective changes in scleral fibers in the posterior pole compared to the primary form (Curtin type I). In addition, the severity of myopic maculopathy and other myopic macular changes may differ between the primary and compound forms of WMPS, considering that the prevalence of legal blindness and the degree of myopic degeneration were different among the staphyloma types in previous studies [7, 8]. In the present study, we compared the clinical features of highly myopic eyes with WMPS according to its structural complexity, between the primary and compound forms.

## Methods

In this retrospective cross-sectional study, we retrospectively reviewed the medical records of highly myopic patients who were examined using ultra-widefield fundus imaging and widefield swept-source optical coherence tomography (OCT) at the Pathologic Myopia Clinic at the Seoul National University Hospital between January 2020 and April 2021. Definition of high myopia was an axial length of longer than 26.5 mm or a myopic refractive error less than − 6.0 diopters, and patients with posterior staphyloma of the wide macular type according to the Ohno-Matsui classification were included. The exclusion criteria were as follows: [1] media opacity that prevented the grading of posterior staphyloma and myopic maculopathy; [2] poor image quality of the swept-source OCT; [3] posterior staphyloma other than the wide macular type; or [4] other retinal disorders including age-related macular degeneration, diabetic retinopathy, and retinal vascular diseases. A previous history of vitreoretinal surgery or intravitreal injection of anti-VEGF was not an exclusion criterion. This study adhered to the tenets of the Declaration of Helsinki. The Institutional Review Board of the Seoul National University Hospital approved the protocol and study design (IRB no. 2003-231-1115) and informed consent was waived because this study poses no more than minimal risks toward participants and has retrospective nature.

A comprehensive ocular examination was performed in all patients, including best-corrected visual acuity (BCVA), slit lamp examination, refractive error, axial length measurement, fundus examination, ultra-widefield fundus imaging, and widefield swept-source OCT. The BCVA was converted to the logarithm of minimal angle of resolution (logMAR) scale for statistical analysis. Axial length was measured using ocular biometry (IOLMaster; Carl Zeiss Meditec, Jena, Germany) by an experienced examiner. Ultra-widefield fundus images were obtained using an Optos 200Tx scanning laser ophthalmoscope (Optos PLC, Scotland, UK) centered on the fovea. Widefield swept-source OCT examination was performed using the PLEX Elite 9000 (Carl Zeiss Meditec, Dublin, CA) with a UHD spotlight B-scan with a 16-mm length and 6-mm depth. In all eyes, horizontal and vertical scan images centered on the fovea and scan images staring from the fovea toward the periphery at the superior, inferior, temporal, and nasal directions were performed to include the edge of posterior staphyloma.

**Fig. 1:**
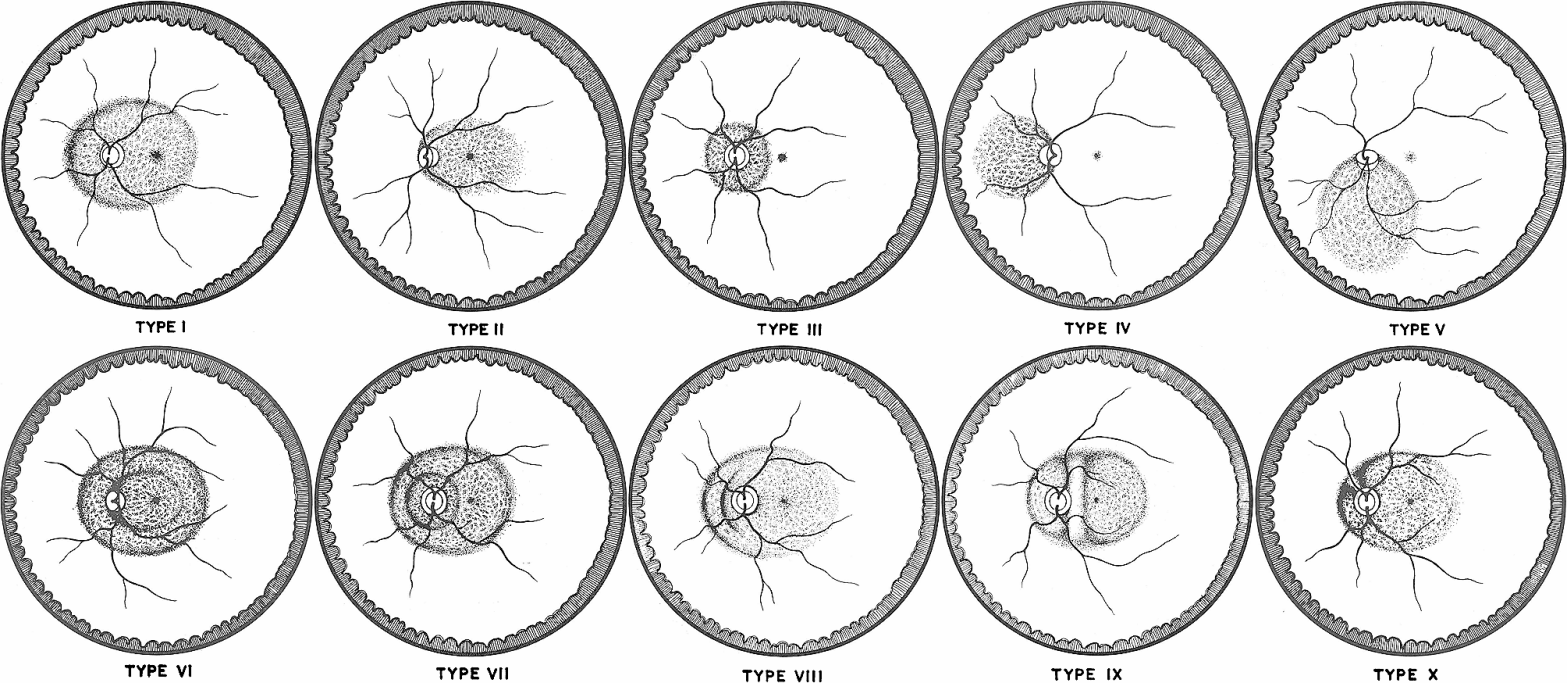
Curtin classification of posterior staphyloma. Types I to V are primary form, while types VI to X are compound form (cited with permission of the American Ophthalmological Society) [7]. As the variants of the primary type I staphyloma, five types (VI to X) of compound staphylomas fall into the same category of type I posterior staphyloma, which is renamed as the wide macular type in the Ohno-Matsui classification [5]

The presence and types of posterior staphyloma were primarily determined by pigmentary abnormalities in the pseudo-color image or reflectance abnormalities in the red or green separation image of ultra-widefield scanning laser ophthalmoscope, which indicate the border of posterior staphyloma [5, 9]. Based on the Ohno-Matsui classification, which considers solely the outermost border of the posterior staphyloma to determine its type, only patients with posterior staphyloma of wide macular type in at least one eye were included. According to the configuration within the staphyloma, WMPS were subclassified into primary and compound forms [7]. In a primary form of WMPS, which corresponds to type I by the Curtin classification, an outpouching with border of singular ellipse encompasses both macula and optic disc without any combined staphyloma. Contrarily, in a compound form, which corresponds to the Curtin types VI (macular staphyloma within type I), VII (peripapillary staphyloma within type I), VIII (tiers or steps across the wall of type I), IX (type I divided into compartments by vertical septum), and X (type I divided into compartments by plication), two or more distinct staphylomas overlap creating multi-tiered depression or are combined side-by-side to create a ridge among them (Fig. 1). For the eyes ambiguous for the presence or type of WMPS, widefield OCT images were also assessed to detect the edge of staphyloma, which is characterized by a gradual thinning of the choroid and an inward protrusion of the sclera [10]. A compound form of WMPS that did not fall within any type of VI to X in the Curtin classification was subclassified as miscellaneous. A WMPS with a simple presence of dome-shaped macula or local excavation of sclera was not considered as the compound form.

Using widefield swept-source OCT, scleral thickness (ST) and choroidal thickness (CT) were manually measured with a caliper provided by the OCT machine at the subfoveal region and 3,000 μm superior, inferior, nasal, and temporal to the fovea center as previously reported [11]. Considering that the dimension on the OCT images is influenced by eyeball length, the actual locations of non-subfoveal measurement were adjusted using a previously reported formula: *t* = 3.382 × 0.01306 × (axial length–1.82) × *s*, where *t* and *s* represent the actual dimensions and measurements on OCT images, respectively [12]. Eyes that underwent vitreoretinal surgeries were not included in the thickness analysis. When analyzing subfoveal ST, eyes with dome-shaped macula were excluded, which was defined as an macular RPE’s inward bulge greater than 50 μm above the bottom of macular curvature.

Clinical and ocular characteristics, such as BCVA, axial length, grading of myopic macular degeneration, frequency of myopic traction maculopathy and active or scarred myopic CNV, CT, and ST, were compared between the eyes with the primary form (Curtin type I) and compound form (Curtin type VI to X) of WMPS. Myopic macular degeneration was graded into four categories according to the Meta-analysis of Pathologic Myopia (META-PM) Study Group classification (category 1, fundus tessellation; category 2, diffuse chorioretinal atrophy; category 3, patchy chorioretinal atrophy; and category 4, macular atrophy) were compared [13]. The myopic traction maculopathy was graded according to the recently introduced Myopic Traction Maculopathy Staging System (MSS), which reflects both anteroposterior and tangential traction [14, 15]. It consists of retinal pattern stage (1, inner or inner-outer maculoschisis; 2, predominantly outer maculoschisis; 3, macular detachment with maculoschisis; and 4, macular detachment without maculoschisis) combined with foveal pattern stage (a, absence of foveal splitting; b, inner lamellar macular hole; and c, full-thickness macular hole) and also includes the presence of associated epiretinal abnormalities and outer lamellar macular hole. In eyes with previous history of vitrectomy to treat myopic traction maculopathy, status of macular traction just prior to the surgery was collected for analysis. Two retinal specialists (CKY and EKL) independently performed grading of myopic maculopathy and posterior staphyloma and measurement of CT and ST in a masked fashion. A senior specialist (UCP) adjudicated any discrepancies, and the measurements from two graders were averaged for statistical analysis. Student’s t-test or Mann-Whitney U test for continuous variables and Chi-squared test or Fisher’s exact test for categorical variables were used to compare the outcomes between the groups. *P* values < 0.05 were considered statistically significant.

## Results

**Fig. 2:**
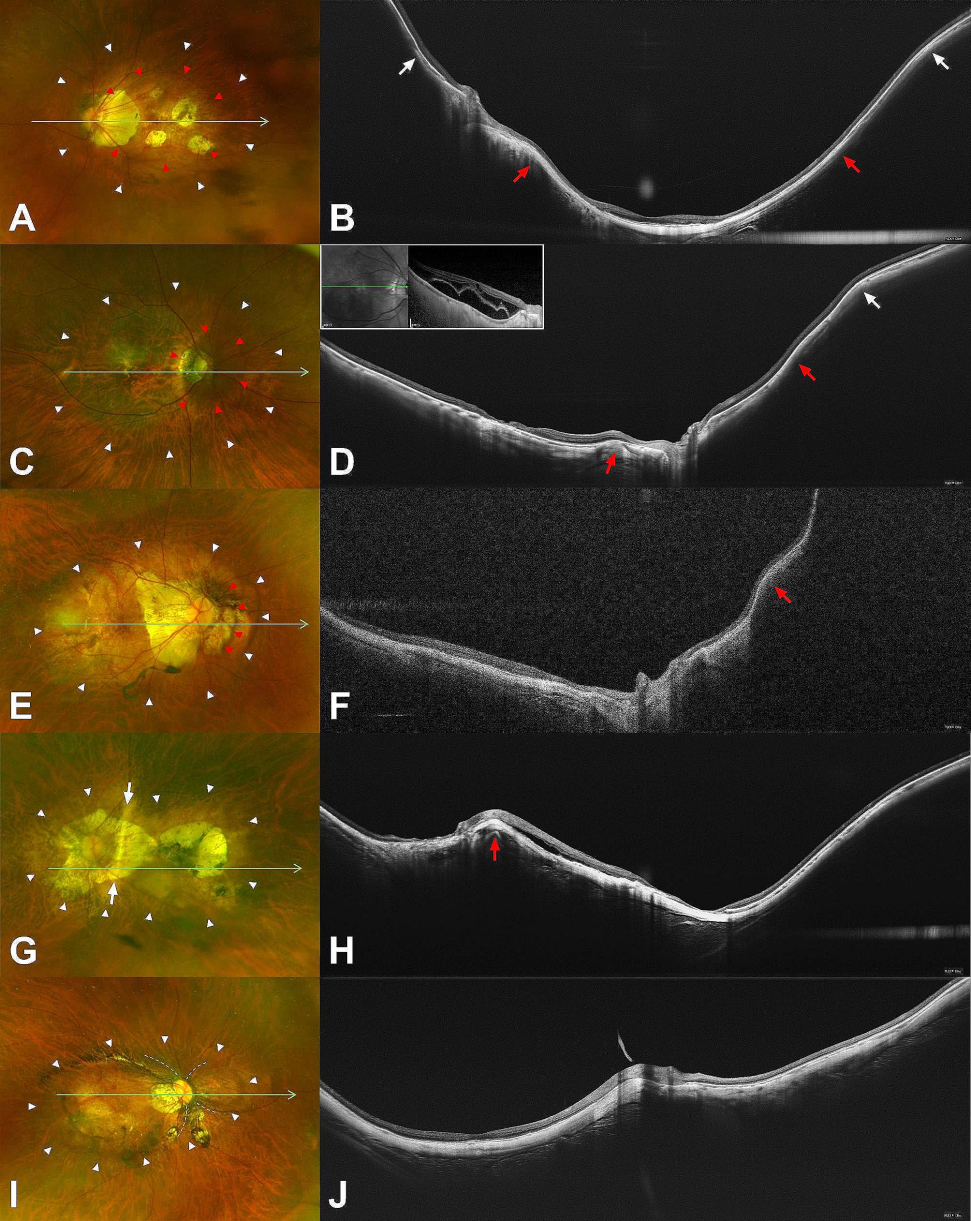
Images of highly myopic eyes with compound form of wide macular posterior staphyloma. The green arrows represent the cross-section through which widefield optical coherence tomography (OCT) scan was performed, and all OCT images are shown on a 1:1 μm scale. (**A, B**) A 72-year-old woman with Curtin type VI staphyloma. The axial length was 32.51 mm and the best-corrected visual acuity (BCVA) was 20/1000. An inner macular staphyloma (red arrowheads) was observed within a primary type I staphyloma (white arrowheads). Macular atrophy and pigmented scar due to previous myopic choroidal neovascularization is observed. Gradual thinning of the choroid is observed at the edges of primary type I staphyloma (white arrows) in the OCT image, but the choroid at the edges of inner macular staphyloma (red arrows) are hardly visible due to patchy atrophy and peripapillary atrophy. (**C, D**) A 76-year-old woman with Curtin type VII staphyloma. The axial length was 28.7 mm and the BCVA was 20/63. A peripapillary staphyloma (red arrowheads) was observed within a primary type I staphyloma (white arrowheads). Gradual thinning of the choroid is observed at the corresponding edges (red and white arrows) in the OCT image. The patient underwent vitrectomy for myopic traction maculopathy with foveal detachment two years ago (inlet). (**E, F**) A 57-year-old man with Curtin type VIII staphyloma. The axial length was 30.13 mm and the BCVA was 20/25. Note the presence of a step (red arrowheads) across the nasal wall of a primary type I staphyloma (white arrowheads). In the OCT, a tiered scleral contour is observed nasal to the optic nerve (red arrow). (**G, H**) A 73-year-old woman with Curtin type IX staphyloma. The axial length was 31.18 mm and the BCVA was count finger. A primary type I staphyloma (white arrowheads) is separated into two parts by a vertical ridge which passes from the upper to the lower border of the staphyloma through temporal side of the optic nerve (white arrows). A protrusion bordering two staphylomas is observed in the OCT image (red arrow). Note macular atrophy and scar change due to previous myopic choroidal neovascularization. (**I, J**) A 66-year-old woman with Curtin type X staphyloma. The axial length was 33.26 mm and the BCVA was 20/500. A primary type I staphyloma (white arrowheads) is separated into two parts by plications extending superior and inferior from the optic nerve (dashed lines)

In this study, 154 eyes (103 patients) with the primary form (Curtin type I) of WMPS and 65 eyes (49 patients) with the compound form (Curtin type VI to X) of WMPS were included. In eyes with compound WMPS, 4 (6.2%), 12 (18.5%), 3 (4.6%), 35 (53.8%), 3 (4.6%), and 8 (12.3%) eyes were subclassified as Curtin staphyloma types VI, VII, VIII, IX, X, and miscellaneous, respectively (Fig. 2). The miscellaneous type of compound WMPS included multiple small staphylomas within the WMPS in five eyes and inner staphyloma not involving the disc or the macula within the WMPS in three eyes. The clinical and ocular characteristics are listed in Table 1. The mean age did not differ between the groups, but eyes with compound WMPS had longer axial length (32.3 ± 2.3 vs. 30.1 ± 2.7 mm; *P* < 0.001) and worse BCVA (0.78 ± 0.67 vs. 0.53 ± 0.53 [Snellen equivalent, 20/121 vs. 20/68]; *P* = 0.004) compared to those with primary WMPS. The proportion of eyes that underwent vitreoretinal surgeries for any reason was significantly greater in eyes with compound WMPS (20/65 [30.8%] vs. 23/154 [14.9%]; *P* = 0.007).

**Table 1:** Baseline characteristics of eyes with primary and compound form of wide macular type posterior staphyloma

Parameters	Form of wide macular type posterior staphyloma		*P*-value
	Primary	Compound	
Number of eyes (%) / patients	154 (70.3%) / 103 patients	65 (29.7%) / 49 patients	-
Mean age, years (range)	66.0 ± 10.6 (39 ~ 96)	64.8 ± 11.0 (39 ~ 85)	0.513
Gender, number of eyes (%) of women	104 (67.5%)	49 (75.4%)	0.264
Mean axial length, mm (range)	30.1 ± 2.7 (24.3 ~ 37.0)	32.3 ± 2.3 (28.0 ~ 37.9)	< 0.001
Mean baseline BCVA, logMAR (range, Snellen equivalent)	0.53 ± 0.53 (-0.18 ~ 2.50, 20/68)	0.78 ± 0.67 (-0.08 ~ 2.50, 20/121)	0.004
Dome-shaped macula	40 (26.0%)	22 (33.8%)	0.253
Any previous vitreoretinal surgery	23 (14.9%)	20 (30.8%)	0.007
Previous vitrectomy for myopic traction maculopathy	16 (10.4%)	10 (15.4%)	0.296
Myopic Macular Degeneration			
Fundus tessellation (category 1)	14 (9.1%)	1 (1.5%)	< 0.001
Diffuse chorioretinal atrophy (category 2)	69 (44.8%)	14 (21.5%)	
Patchy chorioretinal atrophy (category 3)	53 (34.4%)	40 (61.5%)	
Macular atrophy (category 4)	18 (11.7%)	10 (15.4%)	
Myopic Traction Maculopathy (%, number of foveal pattern a/b/c)			
No Myopic Traction Maculopathy	97 (63.0%)	34 (52.3%)	0.332
MSS retinal pattern stage 1	26 (16.9%, 20/4/2)	16 (24.6%, 12/1/3)	
MSS retinal pattern stage 2	23 (14.9%, 18/4/1)	10 (15.4%, 4/6/0)	
MSS retinal pattern stage 3	6 (3.9%, 3/3/0)	2 (3.1%, 0/2/0)	
MSS retinal pattern stage 4	2 (1.3%, 0/0/2)	3 (4.6%, 1/1/1)	
Presence of foveal pattern b or c	16 (10.4%)	14 (21.5%)	0.028
Presence of epiretinal abnormalities (plus sign)	33 (21.4%)	23 (35.4%)	0.031
Presence of outer lamellar macular hole	8 (5.2%)	9 (13.8%)	0.029
Myopic CNV			0.030
No myopic CNV	123 (79.9%)	43 (66.2%)	
Active or inactive scar of myopic CNV	31 (20.1%)	22 (33.8%)	

BCVA = Best-corrected visual acuity; logMAR = logarithm of minimal angle of resolution; MSS = Myopic Traction Maculopathy Staging System; CNV = choroidal neovascularization

Distribution of grading for myopic macular degeneration based on the META-PM classification was different between the groups (*P* < 0.001). Eyes with compound WMPS were more likely to have a higher grade than those with primary WMPS: 9.1%, 44.8%, 34.4%, and 11.7% in eyes with primary WMPS and 1.5%, 21.5%, 61.5%, and 15.4% in eyes with compound WMPS were classified as having fundus tessellation, diffuse atrophy, patchy atrophy, and macular atrophy, respectively. Distribution of MSS stage did not differ between the group (*P* = 0.332), and the proportions of eyes with MSS stage 1 or higher (31/65 [47.7%] vs. 57/154 [37.0%]; *P* = 0.141) and eyes with MSS stage 2 or higher (15/65 [23.1%] vs. 31/154 [20.1%]; *P* = 0.625) were also comparable. However, frequency of foveal pattern of b or higher (inner lamellar macular hole or full-thickness macular hole; *P* = 0.028), associated epiretinal abnormalities (*P* = 0.031), and outer lamellar macular hole (*P* = 0.029) were greater in eyes with compound WMPS. In compound WMPS, proportion of eyes with MSS stage 2 or higher was 25.0%, 41.7%, 0%, 25.7%, 0%, and 0% in Curtin type VI, VII, VIII, IX, X, and miscellaneous (Table 2; *P* = 0.010). Foveal pattern b or higher was also observed only in eyes with Curtin type VI, VII, and IX (25.0%, 41.7%, and 22.9%, respectively; *P* = 0.009). The proportion of eyes that underwent vitrectomy for the treatment of myopic traction maculopathy did not differ between the groups (*P* = 0.296). Active or scarred myopic CNV was observed more frequently in eyes with compound WMPS than in eyes with primary WMPS (22/65 [33.8%] vs. 31/154 [20.1%]; *P* = 0.030).

**Table 2:** Myopic traction maculopathy stages in eyes with compound form of wide macular type posterior staphyloma

	Type VI (*n* = 4)	Type VII (*n* = 12)	Type VIII (*n* = 3)	Type IX (*n* = 35)	Type X (*n* = 3)	Miscellaneous (*n* = 8)	*P*-value
No Myopic Traction Maculopathy	2 (50.0%)	4 (33.3%)	3 (100%)	16 (45.7%)	1 (33.3%)	8 (100%)	0.003^*^ 0.010^**^
MSS retinal pattern stage 1	1 (25.0%)	3 (25.0%)	0 (0%)	10 (28.6%)	2 (66.7%)	0 (0%)	
MSS retinal pattern stage 2	1 (25.0%)	3 (25.0%)	0 (0%)	6 (17.1%)	0 (0%)	0 (0%)	
MSS retinal pattern stage 3	0 (0%)	0 (0%)	0 (0%)	2 (5.7%)	0 (0%)	0 (0%)	
MSS retinal pattern stage 4	0 (0%)	2 (16.7%)	0 (0%)	1 (2.9%)	0 (0%)	0 (0%)	
Presence of foveal pattern a	1 (25.0%)	3 (25.0%)	0 (0%)	11 (31.4%)	2 (66.7%)	0 (0%)	0.009^†^
Presence of foveal pattern b	1 (25.0%)	3 (25.0%)	0 (0%)	6 (17.1%)	0 (0%)	0 (0%)	
Presence of foveal pattern c	0 (0%)	2 (16.7%)	0 (0%)	2 (2.9%)	0 (0%)	0 (0%)	

MSS = Myopic Traction Maculopathy Staging System

*: P-value for retinal pattern stage 1 or higher

**: P-value for retinal pattern stage 2 or higher

†: P-value for foveal pattern b or c

Table 3 shows the comparison of CT and ST according to the complexity of the WMPS. Eyes with previous history of vitreoretinal surgery were not included in the CT and ST analyses. At all five measurement points, the mean CTs in eyes with primary WMPS were significantly greater than in those with compound WMPS (all *P* ≤ 0.004). The posterior scleral border was undetectable on swept-source OCT images in 92 of the 655 measurement points (14.0%) of 131 eyes with primary WMPS and 16 of the 225 measurement points (7.1%) of 45 eyes with compound WMPS (*P* = 0.006); thus, ST was not measurable. The invisible posterior scleral border was significantly more frequent in eyes with primary WMPS than in eyes with compound WMPS at the subfoveal region (*P* = 0.022) but was comparable at other points. At all points, the mean measurable STs in eyes with primary WMPS were greater than in those with compound WMPS (all *P* ≤ 0.015).

**Table 3:** Choroidal and scleral thicknesses in eyes with primary and compound form of wide macular type posterior staphyloma

Parameters	Form of wide macular type posterior staphyloma		*P*-value
	Primary (*n* = 131)	Compound (*n* = 45)	
Choroidal thickness, µm (range)			
Subfoveal	32.4 ± 21.8 (0 ~ 99)	19.1 ± 13.7 (0 ~ 56)	< 0.001
Superior	44.7 ± 32.1 (0 ~ 134)	23.9 ± 21.0 (0 ~ 91)	< 0.001
Inferior	38.5 ± 31.2 (0 ~ 159)	20.8 ± 22.5 (0 ~ 95)	0.001
Temporal	41.8 ± 32.6 (0 ~ 156)	23.3 ± 23.8 (0 ~ 115)	0.001
Nasal	18.5 ± 20.3 (0 ~ 137)	8.7 ± 15.3 (0 ~ 60)	0.004
Scleral thickness, µm (range)			
Subfoveal^*^	315.7 ± 89.4 (108 ~ 638)	209.3 ± 72.1 (103 ~ 387)	< 0.001
Superior	227.7 ± 83.0 (90 ~ 499)	189.7 ± 65.5 (107 ~ 343)	0.015
Inferior	227.6 ± 69.0 (92 ~ 434)	180.4 ± 62.8 (95 ~ 318)	< 0.001
Temporal	216.5 ± 76.8 (83 ~ 456)	175.3 ± 67.4 (86 ~ 360)	0.003
Nasal	266.3 ± 80.7 (77 ~ 467)	205.4 ± 87.8 (63 ~ 488)	< 0.001
Invisible posterior scleral border			
Subfoveal	14 (10.7%)	0 (0.0%)	0.022
Superior	39 (29.8%)	9 (20.0%)	0.204
Inferior	9 (6.9%)	1 (2.2%)	0.245
Temporal	23 (17.6%)	4 (8.9%)	0.163
Nasal	7 (5.3%)	2 (4.4%)	0.813

*: Excluded eyes with dome-shaped macula

## Discussion

In this study, posterior staphyloma of the wide macular type, which is the most frequent type in highly myopic eyes, was subclassified into primary and compound forms according to the configuration within the staphyloma described in the Curtin classification. Eyes with the compound form of WMPS (Curtin type VI to X) had worse visual acuity and were more likely to have more severe myopic macular degeneration and higher frequency of lamellar or full-thickness macular hole associated with myopic traction and active or scarred myopic CNV compared to those with primary form (Curtin type I). In addition, eyeball elongation and thinning of the choroid and sclera were more pronounced in eyes with the compound form of WMPS.

Based on the Ohno-Matsui classification of posterior staphyloma, wide macular type has been reported to be the most predominant, accounting for 74–79% of eyes with posterior staphyloma [5, 9]. In the classification, WMPS includes various forms of staphyloma, not only type I staphyloma but also compound staphyloma of types VI to X in the Curtin classification which are characterized by irregularities within the staphyloma. Although this new classification is more objective and simpler to categorize the morphology of posterior staphyloma, the detailed frequency of the compound form WMPS based on new imaging modalities such as ultra-widefield fundus imaging and widefield OCT has not been reported. In Curtin’s report which was solely based on ophthalmoscopic examination, staphyloma of types VI to X was observed in 125 eyes, while type I was observed in 249 eyes [7]. This proportion was comparable to the present study, which included 65 eyes with compound and 154 eyes with primary WMPS, confirming that the compound form of WMPS may not be uncommon in the highly myopic eyes.

The presence of posterior staphyloma in highly myopic eyes is associated with more severe myopic degeneration in the macula [5, 6], resulting in worse visual acuity. The mechanism for this association was not fully elucidated but decreased ST and CT in eyes with staphyloma suggest that structural or vascular changes resulting from mechanical stretching within the localized outpouching may facilitate the development or progression of myopic maculopathy [11, 16]. It is possible that a localized decrease in scleral resistance, due to alterations in the scleral fiber arrangement and extracellular matrix composition, may lead to the characteristic morphology of staphyloma [17–19]. In this study, eyes with the compound form of WMPS had greater axial length and decreased ST and CT compared to those with primary form. Considering that the compound form of WMPS has additional outpouchings within the primary staphyloma, the pathogenetic hypothesis of posterior staphyloma compared to its absence may also apply to the compound WMPS compared to its primary form. In eyes with compound WMPS, there seems to be an area with more decreased scleral resistance even within the primary outpouching, possibly due to topographical heterogeneity in the scleral fiber architecture.

In this study, eyes with compound WMPS had higher grades of myopic macular degeneration and higher frequency of neovascularization and foveal change associated with myopic macular traction. More severe myopic macular changes in these eyes may be associated with greater structural changes in the posterior eyeball wall, resulting in increased mechanical stress on the macula. In addition, some types of compound WMPS (Curtin type VI, VII, and IX) were associated with higher likelihood of myopic traction maculopathy than other types, and this suggests that traction forces exerted at the macula may be different according to the scleral configuration within the staphyloma. This assumption is supported by a previous study by Hsiang et al. which showed a deeper staphyloma is associated with more severe myopic degenerative changes [8]. Further, they could evaluate long-term change of posterior staphyloma in 10 eyes for longer than 20 years. All three eyes that experienced morphological changes in the staphyloma from primary to compound form (Curtin type II to IX) showed progression of myopic degeneration during follow-up, while only two of seven eyes that remained as primary staphyloma showed progression. Taken together, results of the present study suggest that wide macular type of posterior staphyloma in the Ohno-Matsui classification has a great variety of morphology within its outermost border. Worse visual prognosis associated with more severe structural deformation in eyes with the compound form of WMPS implies that it may be a more advanced form of WMPS and more attention should be paid to the development and progression of myopic macular complications. It may be classified separately as a severe clinical entity with poor prognosis, like “wide macular plus” or “compound wide macular” type to differentiate from primary wide macular type.

**Fig. 3:**
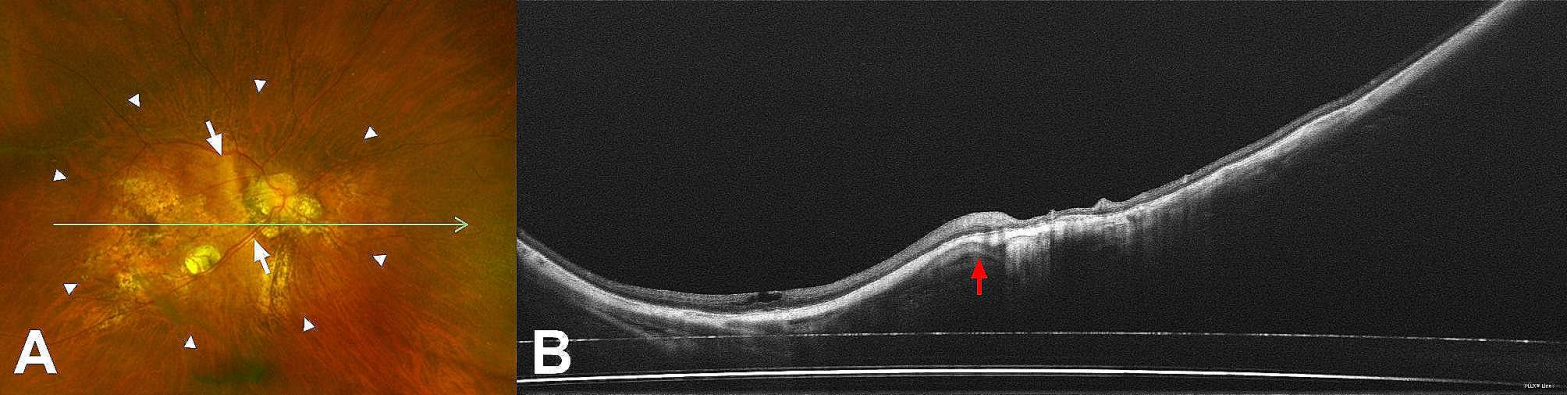
(**A**) Ultra-widefield fundus image of an 81-year-old woman shows wide macular type posterior staphyloma (white arrowheads) and a vertical septum-like structure temporal to the optic nerve (white arrows). (**B**) Widefield optical coherence tomography scan image through the green arrow in A shows that it is not a real protrusion (red arrow) and this wide macular type posterior staphyloma corresponds to a primary type I staphyloma

In this study, Curtin type IX was most frequent in eyes with compound WMPS. This was in accordance with the results of Hsiang et al. [8] although they reported no cases with other types of compound WMPS. However, in Curtin’s report, types VIII and X were observed with a similar frequency to type IX. The discrepancy in the prevalence of each type of compound form of WMPS in these studies may suggest that assessment of staphyloma configuration based on binocular stereoscopic ophthalmoscopy may be imprecise. In this study, the types of WMPS were determined using ultra-widefield fundus image and widefield OCT, and this may have enabled a more accurate evaluation of the posterior eyeball structure [10, 20]. For example, vertical septum-like structure observed temporal to the optic nerve is not always a real protrusion but can be due to peripapillary choroidal thinning, and the horizontal OCT image traversing the optic nerve can reveal the true type of posterior staphyloma (Fig. 3).

There are some limitations to this study. Firstly, it had a cross-sectional design and did not reveal longitudinal changes in the morphology of primary and compound WMPS. Thus, the development sequence among each staphyloma constituting the compound WMPS, whether the primary and inner secondary staphylomas develop simultaneously or inner staphyloma develops later within the primary staphyloma, could not be established based on our results. Secondly, the causal or temporal relationship between the structural changes in the eyeball and the myopic maculopathy progression was difficult to determine. Long-term follow-up is required to reveal the morphological changes in the compound form of WMPS to elucidate its influence on myopic alterations in the macula and visual acuity. Thirdly, choroidal thickness has a diurnal variation but the time of choroidal thickness measurement was not taken into account in this study. Finally, classification of different types of WMPS based on ultra-widefield fundus image and widefield OCT may be less objective than using 3D-MRI, which was not performed in this study.

## Conclusions

In conclusion, this study demonstrates that the compound form of WMPS, which has additional scleral protrusion within the primary WMPS, is associated with more severe myopic macular changes and worse visual acuity compared to the primary form of WMPS. Choroidal and scleral thicknesses were more decreased in the compound form of WMPS, which may imply that the biomechanical properties of the sclera are more altered than those of the primary WMPS. Due to the worse visual prognosis associated with more severe structural deformation, compound WMPS should be considered as a more advanced form of staphyloma, requiring more attention to the myopic macular complications.

## Data Availability

The datasets used and/or analysed during the current study are available from the corresponding author on reasonable request.

## Abbreviations

CNV: Choroidal neovascularization
WMPS: Wide macular posterior staphyloma
OCT: Optical coherence tomography
BCVA: Best-corrected visual acuity
logMAR: Logarithm of minimal angle of resolution
ST: Scleral thickness
CT: Choroidal thickness
META-PM: Meta-analysis of Pathologic Myopia
